# Uterine Carcinosarcoma with Alpha-Fetoprotein-Producing Hepatoid Component: A Case Report and Literature Review

**DOI:** 10.1155/2018/3972353

**Published:** 2018-06-11

**Authors:** Joshua J. X. Li, Jacqueline H. S. Lee, Vicky T. C. Chan, Mei-yung Yu

**Affiliations:** ^1^Department of Anatomical and Cellular Pathology, Prince of Wales Hospital, The Chinese University of Hong Kong, Hong Kong; ^2^Department of Obstetrics and Gynaecology, Prince of Wales Hospital, The Chinese University of Hong Kong, Hong Kong; ^3^Department of Clinical Oncology, Prince of Wales Hospital, The Chinese University of Hong Kong, Hong Kong

## Abstract

A 67-year-old woman presented with postmenopausal vaginal bleeding. Full body imaging demonstrated an intrauterine mass with deep myometrial invasion but no nodal or other metastatic disease. Uterine curettage was performed. Histologically, the tumor was an endometrioid adenocarcinoma with sarcomatous element and a hepatoid component, the latter was immunohistochemically positive for alpha-fetoprotein, HepPar-1, and arginase-1. The patient underwent total abdominal hysterectomy and bilateral salpingo-oophorectomy. Serum alpha-fetoprotein level decreased from 31896 ug/l preoperatively to 2063 ug/l postoperatively. Eight weeks later, a rise in serum alpha-fetoprotein was detected, and a biopsy-proven vaginal recurrence was diagnosed. Palliative chemotherapy led to tumor shrinkage and a concurrent decrease in the serum alpha-fetoprotein level. A rise in serum alpha-fetoprotein, refractory to second-line chemotherapy, was accompanied by subsequent development of ureteric obstruction, ascites, and radiological evidence of peritoneal metastases. This is an unusual case of uterine carcinosarcoma with an alpha-fetoprotein-producing hepatoid adenocarcinoma component. Serum alpha-fetoprotein level corresponds to disease recurrence and progression.

## 1. Introduction

Hepatoid adenocarcinomas are rare tumors with histologic appearance resembling malignant hepatocytes with expression of alpha-fetoprotein (AFP), both immunohistochemically and serologically. They have been reported in various sites including the lung, colon, renal pelvis, stomach, gallbladder, and the female genital tract (endometrium, ovary) [1–7]. To date, only four cases of uterine carcinosarcomas with hepatoid adenocarcinomas have been reported [8–10]. We present a case of AFP-producing uterine carcinosarcoma with hepatoid adenocarcinoma, with a review of the literature and emphasis on the role of serum AFP in monitoring disease progression.

## 2. Case Report

**Figure 1:**
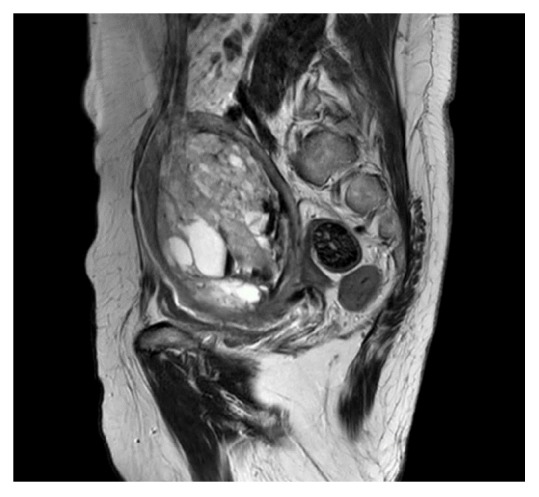
Large uterine tumor with deep myometrial invasion (MRI pelvis, T2 weighted sagittal).

**Figure 2:**
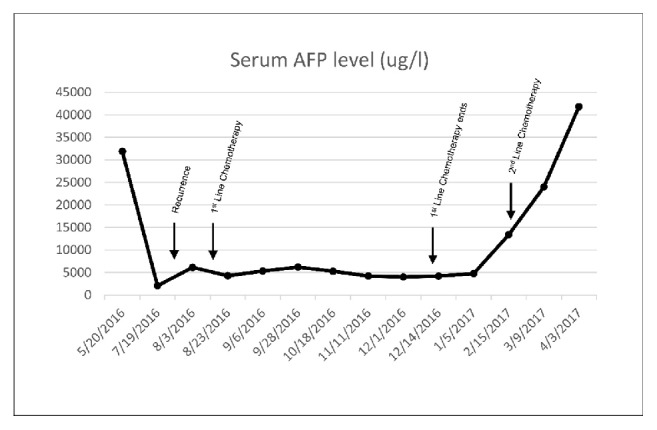
Serum AFP progression.

A 67-year-old woman, gravida 1, para 1, with a medical history of psoriasis and bipolar affective disorder, presented with postmenopausal vaginal bleeding. Physical examination found an 18-week sized uterus without palpable groin lymph nodes. Both adnexa were unremarkable. Magnetic resonance imaging (MRI) of the pelvis and computed tomography (CT) with contrast of the abdomen and thorax demonstrated a localized anterior intrauterine mass with deep myometrial invasion. There was no pelvic or inguinal lymphadenopathy [Figure 1]. The liver was normal in size and outline, with no mass lesion demonstrated on contrast CT. All other intra-abdominal organs were unremarkable. Histologically, the uterine curettage showed carcinosarcoma composed of mixed endometrioid adenocarcinoma, chondrosarcoma, and a hepatoid component. The hepatoid component consisted of trabeculae of polygonal cells with moderate amount of eosinophilic cytoplasm, round to oval nuclei and distinct nucleoli, histologically reminiscent of hepatocellular carcinoma. Immunohistochemically (IHC), the hepatoid tumor cells are positive for AFP, HepPar-1, and arginase-1. Preoperative hepatitis B virus surface antigen was negative and liver function was normal. The patient underwent total abdominal hysterectomy and bilateral salpingo-oophorectomy. Serum alpha-fetoprotein (AFP) dropped from 31896 ug/l preoperatively to 2063 ug/l postoperatively [Figure 2]. Carbohydrate antigen 125 (CA125) level was normal.

**Figure 3:**
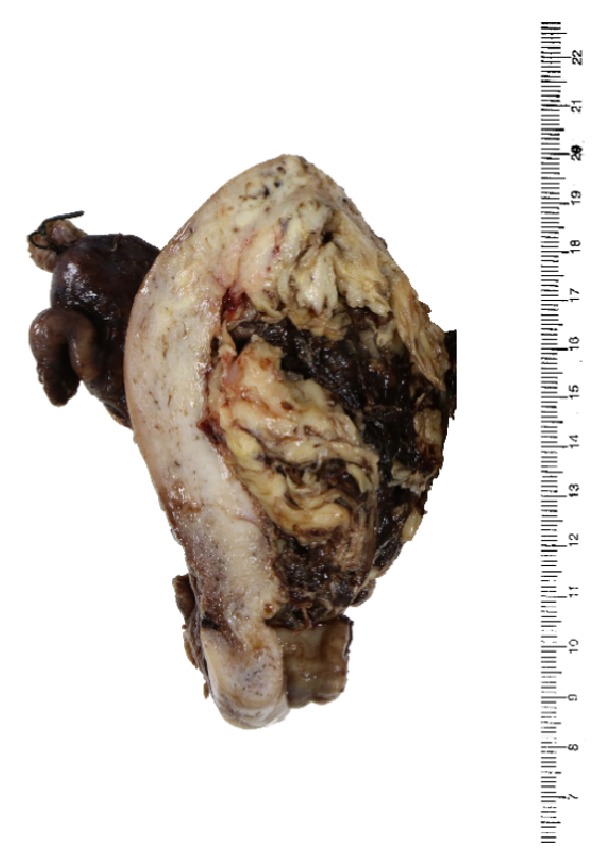
Cut surface of the resected uterus.

**Figure 4:**
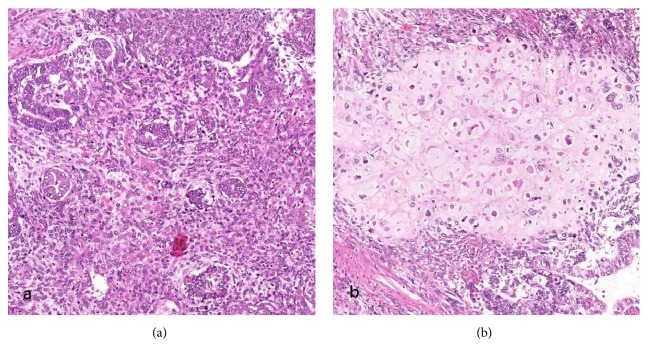
(a) Endometrioid adenocarcinoma components (H&E); (b) chondrosarcomato id components (H&E).

**Figure 5:**
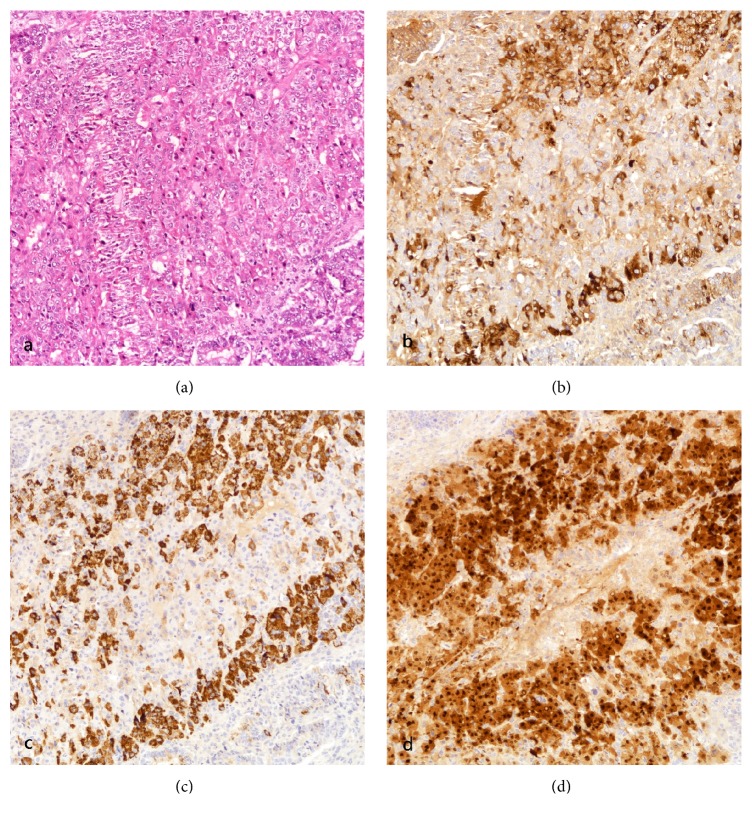
(a) Hepatoid adenocarcinomatous components in trabeculae, polygonal cells with eosinophilic cytoplasm (H&E); (b) AFP (Dako A0008, 1:2000, citrate at 98°C for 30 min); (c) HepPar-1 (Dako M7158, 1:900, EDTA at 98°C for 32 min); (d) arginase-1 (Cell Marque 380R-16, 1:400, EDTA at 98°C for 30 min).

The resected specimen weighted 575 g and measured 11.0 x 9.5 x 8.0 cm with an anterior exophytic tumor measuring 7.5 x 6.0 x 4.0 cm with a tan cut surface [Figure 3] and detached hemorrhagic fragments. Microscopically, the tumor involved the outer half of the myometrium without extension to the cervix or the vagina. Bilateral ovaries were involved. Extensive lymphovascular permeation was seen. Histologic findings were those of a carcinosarcoma with endometrioid adenocarcinoma (20%), hepatoid adenocarcinoma (20%), and sarcomatous components consisting of chondroid (10%) and spindle cell components (50%) [Figure 4]. IHC staining was repeated and the profile was the same as the curettage specimen [Figure 5].

**Figure 6:**
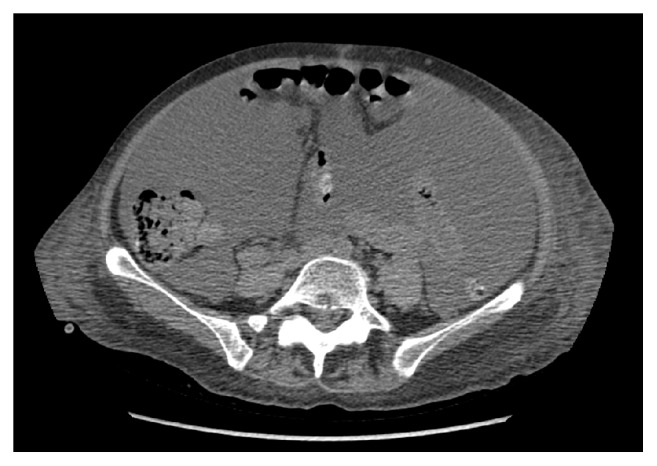
Liver metastasis (CT abdomen and pelvis, noncontrast transverse).

The postoperative clinical course was uneventful, and she was planned to receive taxotere and cyclophosphamide (TC) as adjuvant chemotherapy. Before adjuvant therapy was initiated, a tumor was found in the residual vaginal canal on 8-week postoperative follow-up clinical examination, and biopsy confirming histological recurrence. The patient proceeded to chemotherapy (TC), and the serum AFP level showed a decrease from 6152 ug/l to 4288 ug/l after the first cycle. Serological response was sustained throughout the six cycles of chemotherapy, with a postchemotherapy serum AFP level of 4770 ug/l [Figure 2]. Follow-up CT of the thorax, abdomen, and pelvis did not show any radiological evidence of disease. A subsequent rise of serum AFP to 13409 ug/l was detected 1 month after completion of chemotherapy and the patient was started on Adriamycin. Serum AFP did not show response and was further elevated to 41826 ug/l after cycle two [Figure 2], accompanied by the development of ureteric obstruction, ascites, and radiological evidence of liver and peritoneal metastases [Figure 6]. The patient succumbed 11 months postoperation.

## 3. Discussion

Hepatoid adenocarcinomas have been reported in lung, colon, renal pelvis, stomach, gallbladder, and the female genital tract (endometrium, ovary) [1–7]. The hepatoid adenocarcinoma component usually expresses AFP and some may also express HepPar-1 [1, 11], arginase-1 [11], and glypican-3 [11]. An elevated serum AFP is usually detected in hepatoid adenocarcinoma of various sites, including uterus [1–3, 6–9].

Heterologous sarcomatous components, including most commonly rhabdomyoid [12], chondroid, and osteoid differentiation, are commonly found in uterine carcinosarcomas and are associated with poor prognosis [13]. However, due to the rarity of cases, the impact of a hepatoid epithelial component on the clinical outcome of uterine carcinosarcoma has been poorly documented. Hepatoid adenocarcinoma of the ovary has similar outcomes to other ovarian carcinomas [7], whereas gastric hepatoid adenocarcinoma was reported to be more prone to lymph node and liver metastases, with a poorer overall survival compared to ordinary gastric adenocarcinoma [14].

Literature review yielded only four cases of uterine carcinosarcoma with hepatoid adenocarcinoma component [8–10], all occurring in postmenopausal women with increased serum AFP level and AFP positive staining tumor. Three case reports included clinical outcomes [9, 10] (Table 1). In two cases with favorable outcome, the patients were alive without disease at 12- and 24-month follow-up, respectively, after receiving adjuvant chemotherapy and had attained and sustained normal postoperative serum AFP levels [9, 10]. The single case with reported disease-related mortality was an 82-year-old patient who developed lung metastasis, accompanied by a rise in serum AFP, and succumbed 12 months after her operation [10]. Including the current case, locoregional or distant progression of the disease was noted in 50% of the cases (2 of 4 cases) over a short period of time (12 months or less) attesting to the aggressiveness of this tumor. In all cases it appeared that serum AFP level was a sensitive and accurate marker for monitoring disease progression, with a dropping level indicating response to therapy and rising level auguring disease progression. Despite the short follow-up period for all cases, attainment of low level implies disease remission. Normalization of postoperative serum AFP has been observed in other organ sites [3, 6, 7]. In the relatively well-studied gastric hepatoid adenocarcinoma, a case was reported [15] with a rebound of serum AFP along with CT-proven lung metastasis at 5 months postoperation; the patient succumbed at 18 months with progression of disease.

The current case highlighted the aggressive clinical course of this rare tumor, with a propensity of local progression over short time span. Furthermore, the disease progression can be monitored by serum AFP level. Increased level indicated possible recurrence/metastases, and failure to achieve normal AFP level also indicated compromised outcome.

## Figures and Tables

**Table 1:** Previously reported cases of uterine carcinosarcoma with hepatoid adenocarcinoma component.

	Case 1 (Takano et al.)	Case 2 (Kawaguchi et al.)	Case 3 (Kawaguchi et al.)
Age	63	63	82

Components	Endometrioid adenocarcinoma Hepatoid adenocarcinoma Spindle cell sarcoma	Hepatoid adenocarcinoma Rhabdomyosarcoma	Endometrioid adenocarcinoma Hepatoid adenocarcinoma Spindle cell sarcoma

IHC	AFP +	AFP +	AFP +

Pre-op AFP	5060 ng/ml	10,131 ng/ml	401 ng/ml

Post-op AFP	< 10 ng/ml	< 20 ng/ml	< 20 ng/ml

Adjuvant	Paclitaxel + carboplatin	Paclitaxel + carboplatin	None

Outcome	Disease free at 12 months	Disease free at 24 months	Serum AFP rise Died of lung recurrence at 12 months

## Data Availability

Data sharing not applicable to this article as no datasets were generated or analysed during the current study.
